# Growth, structure, and luminescence properties of novel silica nanowires and interconnected nanorings

**DOI:** 10.1038/s41598-017-10933-7

**Published:** 2017-09-05

**Authors:** Xin Min, Minghao Fang, Haitao Liu, Yan’gai Liu, Xiaowen Wu, Zhaohui Huang

**Affiliations:** Beijing Key Laboratory of Materials Utilization of Nonmetallic Minerals and Solid Wastes, National Laboratory of Mineral Materials, School of Materials Science and Technology, China University of Geosciences (Beijing), Beijing, 100083 P. R. China

## Abstract

Novel silica nanowires and interconnected nanorings were firstly synthesized on a graphite paper by typical thermal catalytic chemical vapor deposition method, using silicon and carbon black powders as raw materials. The field emission scanning electron microscopy, energy dispersive x-ray spectroscopy, Fourier transform infrared spectroscopy, X-ray photoelectron spectroscopy, and transmission electron microscopy were used to investigate the composition and structure characterization, which indicates that the silica nanowires and interconnected nanorings were amorphous. The growth of the as-prepared silica nanowires and interconnected nanorings was related to the vapor-liquid-solid mechanism, but the nanowire-ring structure may be due to the polycentric nucleation and periodic stable growth with gradual direction changes. The room temperature photoluminescence emission spectrum showed that the silica nanostructures emitted strong blue light at 460 nm, resulting from the combination of neutral oxygen vacancy (≡Si-Si≡) and selftrapped excitons. The as-synthesized novel silica nanowires and interconnected nanorings could be a potential candidate for applications in future light-emitting diodes and optoelectronic nanodevices.

## Introduction

One dimensional (1D) nanostructured materials have attracted worldwide intense attention due to their novel optical and electrical properties, and owned potential applications in the integrated energy storage, sensors, photonics, and electronics devices^1–5^. As known to all, the properties and potential applications for nanomaterials were always affected by their morphology and structure. Therefore, most studies recently, on one side, were focused on the development of nanostructures (for example, ZnO, Al_2_O_3_, SiC, InN, and In_2_Ge_2_O_7_) with multifarious morphologies, such as nanowires, nanobelts, nanorods, nanotubes, nanocables, nanorings, and nanochains^6–12^. And on the other hand, various methods also have been applied to assemble the as-prepared nanostructures into desired architectures for the practical needs of nanophotonics and nanoelectronics, and reported many exciting nano-micro-scale structures for function oxides (MgO, ZnO, SnO, and SiO_x_), like fish bones, combs, dendrite spines, bridges, and windmills^13–17^.

Among these functional nanostructures, silica nanowires are important optical materials for potential application in low dimensional wave-guides for future integrated optical devices and high-resolution optical heads of scanning near-field optical microscopes, due to their intense and stable emission^18, 19^. Similarly, the observed luminescence properties of silica nanostructures are significantly different in various morphologies and also different processing methods^20^, such as the laser-ablated silica nanowires with two strong blue bands at 420 and 470 nm^21^, the thermal evaporated silica nanowires with strong green emission at 540 nm^22^, the chemical vapor deposition (CVD) jellyfish-like nanowires with a blue emission band at 415 nm^18^, as well as the spring-shaped, fishbone-shaped, frog-egg-shaped, and pearl-shaped silica nanowires with different properties and corresponding applications^23–27^. For the development of classical optical waveguides, the different cross sections would result in different transverse optical modes^28^. Thus, various structures and shapes for waveguides were studied and reported recently, such as Y-branching^29^, star type^30^, and ring structure^31^. Therefore, the preparation of silica nanowires with novel structures is now considered as an interesting and meaningful research direction.

In this paper, the novel silica interconnected nanorings were prepared and reported for the first time. Silica powder and coke powder were used as raw materials, and aluminum nitrate acted as a catalyst. The growth mechanism for the silica nanowire (NWs) and interconnected nanorings (ICNRs) was discussed in detail. The photoluminescence (PL) spectrum was investigated to clarify the optical properties of as-prepared samples. We think this study could provide a new way to synthesize novel morphology nanostructures of silica and other oxide or non-oxide materials for the application of new generation nano-devices with tunable photoelectric performance.

## Results

**Figure 1:**
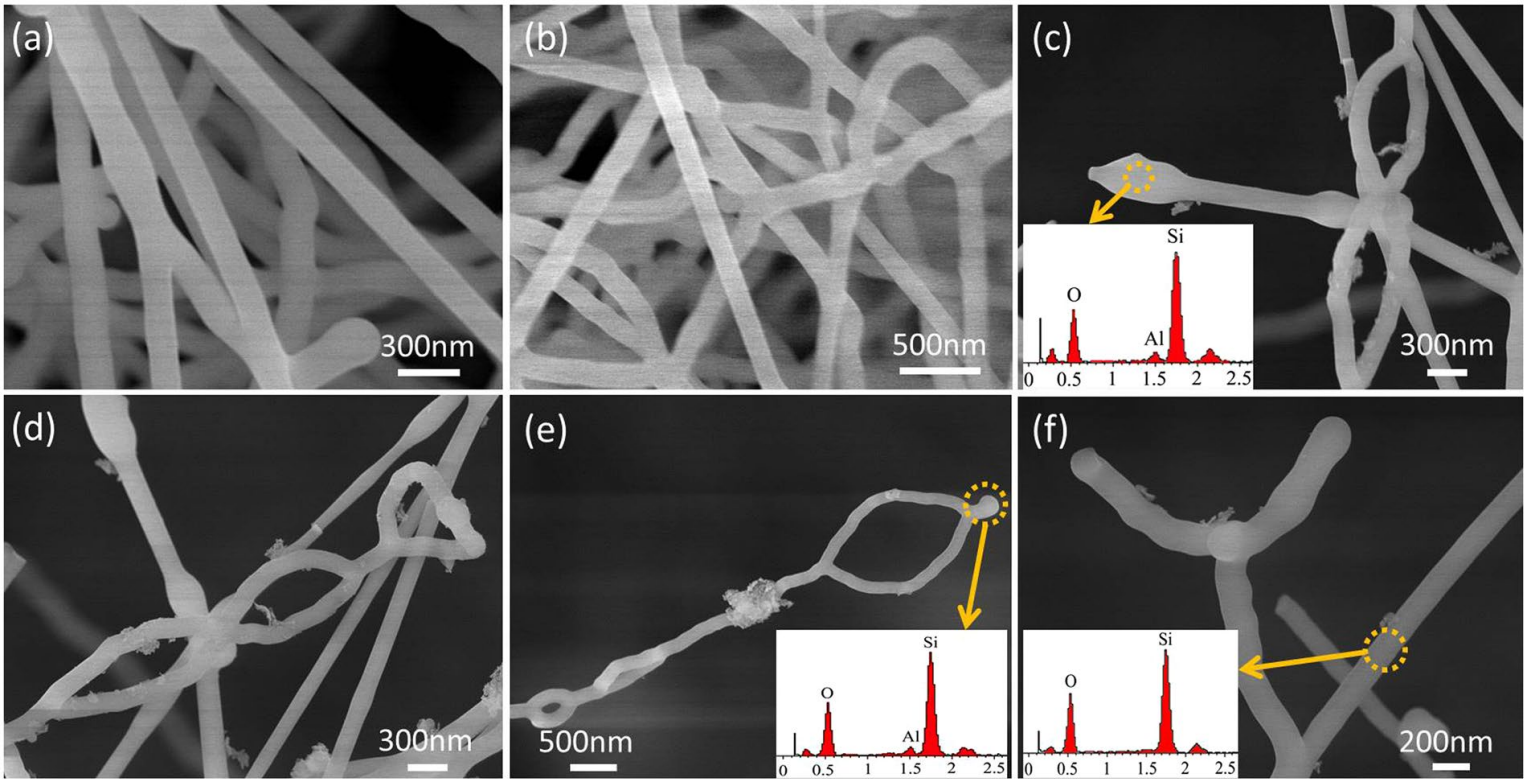
(**a**–**f**) FESEM images of the silica NWs and ICNRs samples prepared on the graphite paper, and the insets of (**c**,**e**,**f)** are EDS spectra taken from the marked areas in corresponding images.

Figure 1(a–f) show the FESEM images of the as-prepared silica 1D nanostructures synthesized on the graphite paper via a simplified CVD method, using silica and coke powders as raw materials, Al(NO_3_)_3_ as catalyst, and argon as flowing gas. The morphology results indicated that of these silica nanostructures were mainly composed of NWs and ICNRs. For here, the amount of the ICNRs nanostructures in the products is about 50%, and this study is the first time to report the novel structure so far. Both the wire size of nanostructures is uniform and its diameter is about 200 nm. According to the separated ICNRs images shown in Fig. 1(d,e), most of the silica nanorings nanostructures are oval-shaped, and the ring size is not uniform. However, all of the nanorings are connected by nanowires and formed the ICNRs structures finally. Figure 1(f) depicts a Y-branch silica nanowire, which can be considered as the intermediate structure of the nanorings. The inset EDS spectrum in Fig. 1(f) revels that the composition of the NW and ICNR nanostructures only consist of Si and O elements. But the EDS spectra inset in Fig. 1(c,d) exhibits that the top of the nanowires composition contain Si, O, and Al elements, which means that Al might provide an initial site for the nucleation and growth of silica nanostructures.

**Figure 2:**
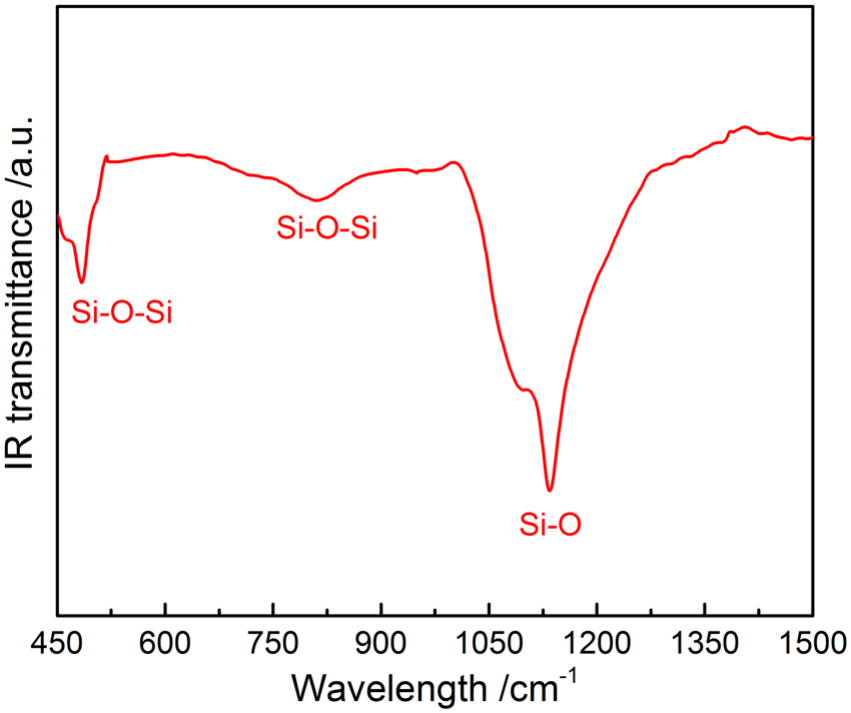
fx1FT-IR spectrum of the as-prepared nanostructures.

**Figure 3:**
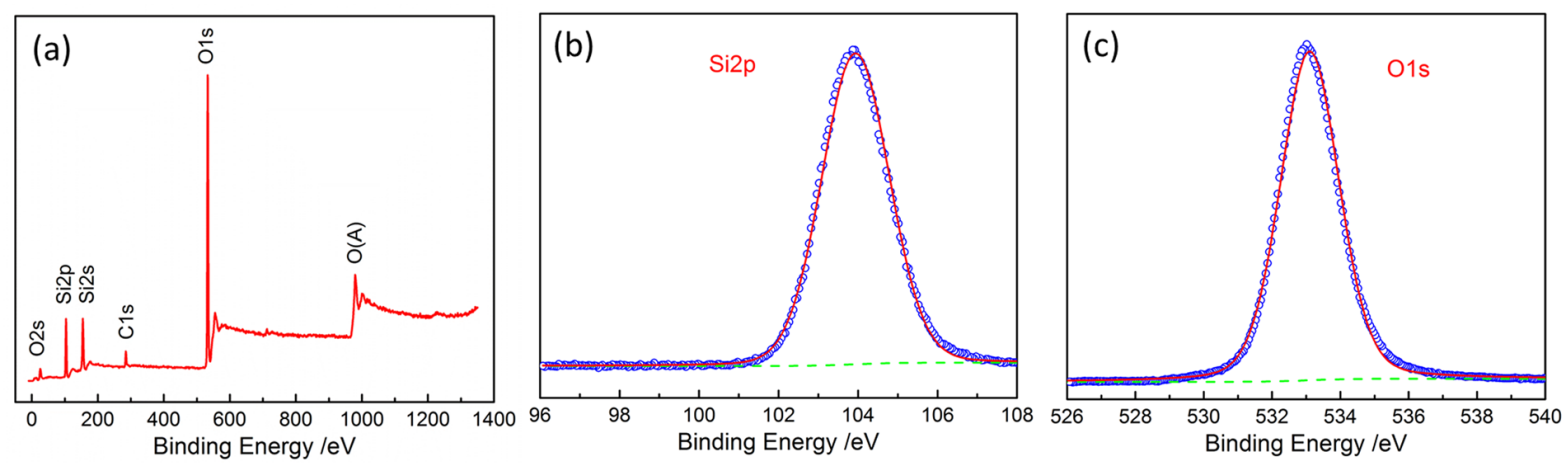
(**a**) XPS survey scan spectrum, (**b**) Si 2p spectra, and (**c**) O 1s spectra of the as-synthesized silica nanostructure products.

Figure 2 depicts the FT-IR spectroscopy pattern of synthesized silica NWs and ICNRs products. As is shown in the spectrum, the two peaks centered at 485 cm^−1^ and 812 cm^−1^ are attributed to the Si-O-Si stretching vibration, and the peak at about 1135 cm^−1^ is correspond to the Si-O stretching vibration of amorphous SiO_x_. Moreover, XPS data was also used to confirm the elemental makeup and the oxidation state of the as-prepared nanostructures. As is shown in Fig. 3(a), the survey scan spectrum indicates that only Si and O core levels was observed, besides the C 1s standard peak at 284.79 eV. The two high-resolution XPS spectra of Si 2p and O 1s as shown in Fig. 3(b,c) are confirmed to be both good symmetrical peaks after deconvolution analyzing, centered at 103.88 eV and 533.04 eV, respectively. These two peak positions are well fitted with the literature binding energy values of Si 2p and O 1s for SiO_2_^32, 33^. No any other obvious Si XPS peaks are observed here. The atom ratio of Si (30.31%) and O (61.24%) is calculated to be 1:2.02, which is just a little higher than the ideal proportion of Si and O elements for SiO_2_ due to some atomic oxygen adsorbed on the surface. Therefore, it can be concluded the composition as-prepared samples are silica products.

**Figure 4:**
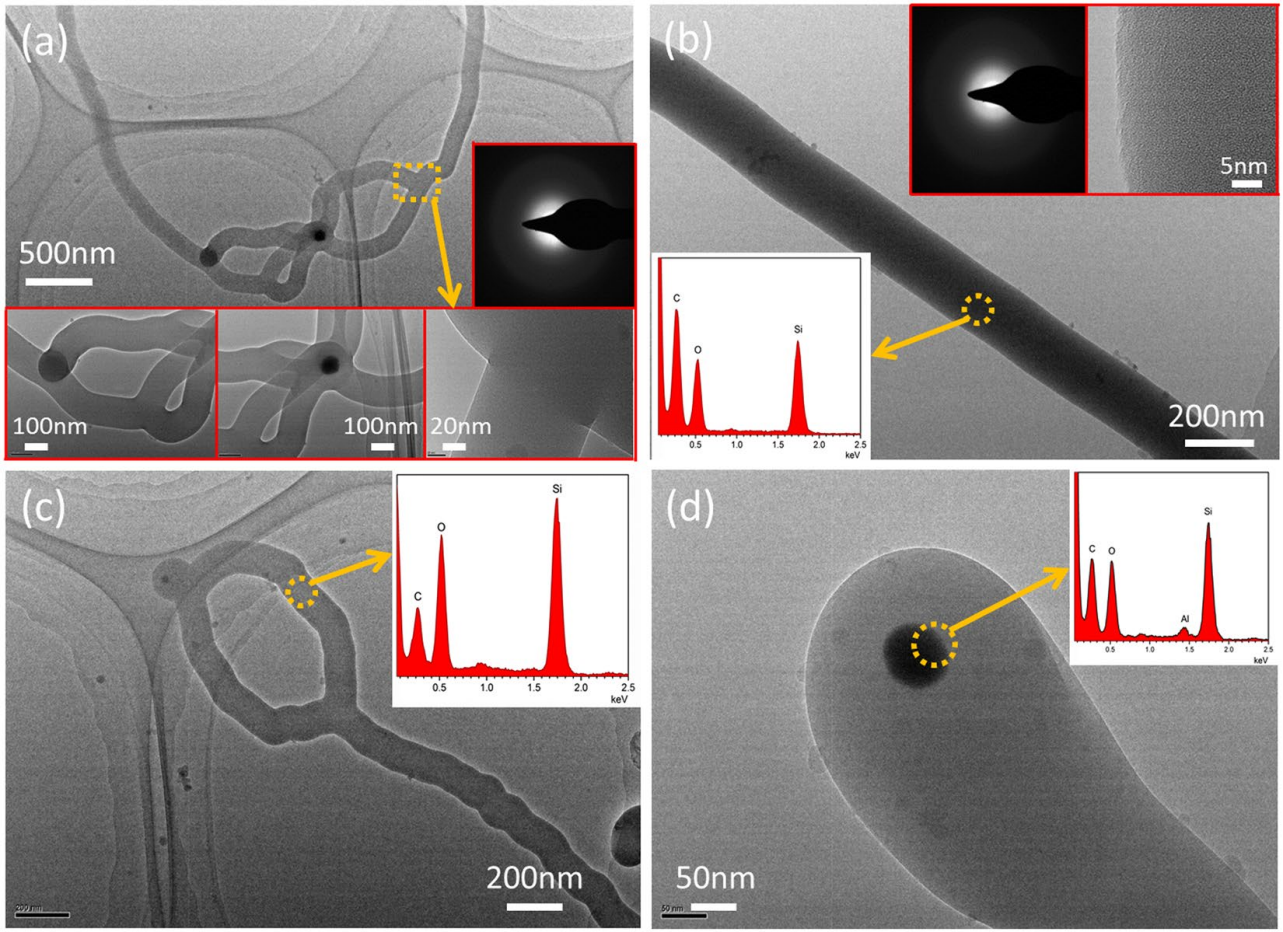
Typical TEM image of the ICNRs (**a**), single NW (**b**), and independent ICNR (**c**). Insets in (**a**) and (**b**) depicts the high magnification TEM, HRTEM, and electron diffraction images. (**c**) Conventional TEM micrograph of the apex of the as-synthesized silica structures. The EDS spectra of the selected areas inset in the corresponding images in figure (**b**–**d**).

Further investigation on the internal microstructure of the as-fabricated nanostructures was initiated by TEM equipped with EDS, and the details were presented in Fig. 4. Figure 4(a) depicts the typical TEM images of the silica NW with interconnected nanorings. The nanowire has about 200 nm in diameter and few micrometers in length, and their surface is smooth. The high magnification TEM and HRTEM images of the nanorings and junctions, shown in the insets of Fig. 4(a), indicates that the junctions of nanoring and nanowire are formed naturally during the growth of interconnected nanoring structures as opposed to the simple overlapped wires. Figure 4(b) shows the TEM image of an individual silica nanowire, processing a similar size with the ICNR structures in Fig. 4(a). The HRTEM (inset at the top-right corner of Fig. 4(b)) analysis indicates that the nanowire is amorphous. Meanwhile, the electron diffraction micrographs inset in Fig. 4(a,b) also provide the evidence for their amorphous structure. Figure 4(c) indicates a nanoring at the end of the nanowire to form the novel nanowire-rings structure, which has a smaller diameter about 150 nm. The inset EDS results in Fig. 4(b) and (c) recorded from the marked areas reveals that both the NW and ICNR are composed of Si and O atoms with the ratio about 2:1. Figure 4(d) exhibits the magnification TEM image and the EDS data of the apex of the NWs. As it can be seen, a small dark droplet is at the top of the NW, and the element composition is detected to be Si, O, and Al, which suggests that the Al plays an important role in initial nucleation and further growth of the amorphous silica NWs and ICNRs.

**Figure 5:**
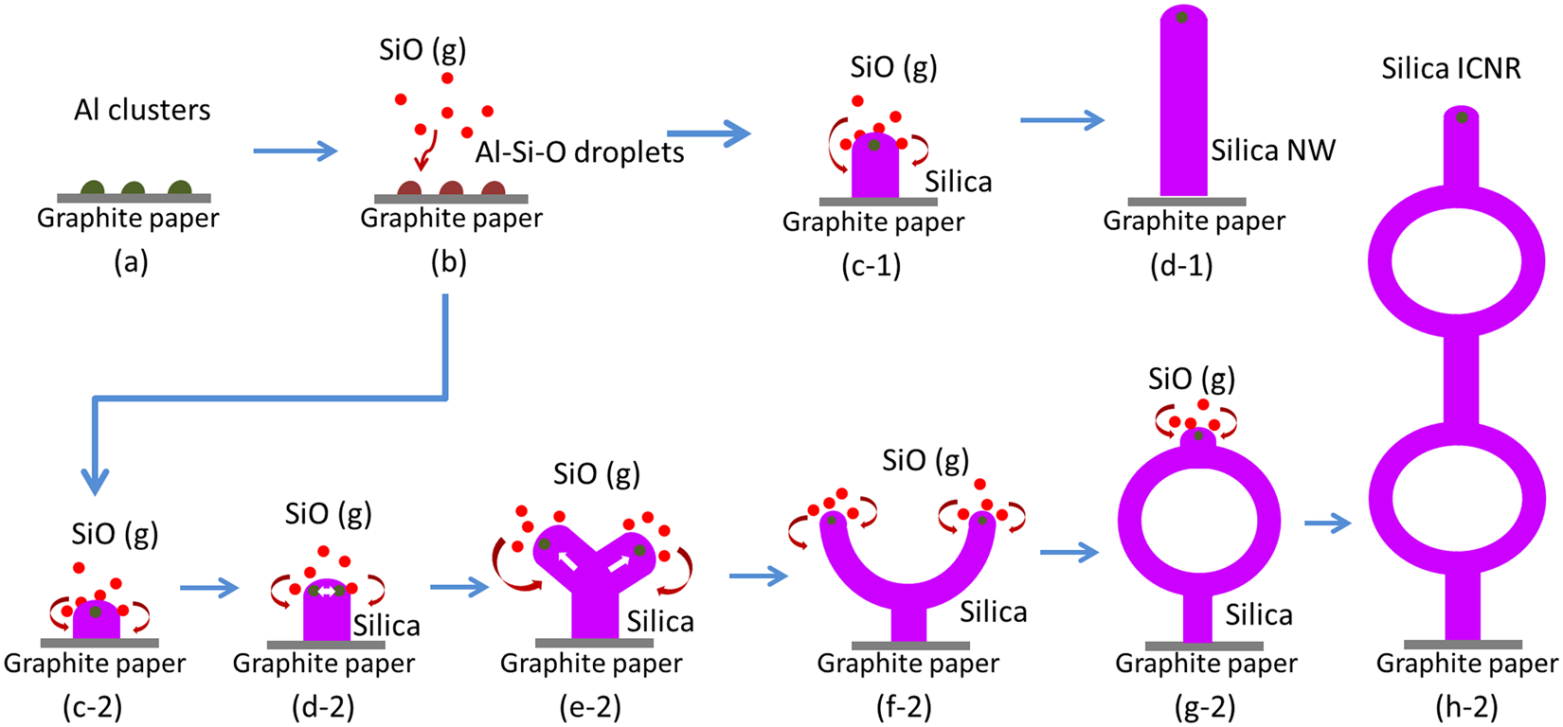
Newly proposed VLS growth model for silica NWs and ICNRs. (**a**) the formed Al nanoparticles, (**b**) Absorption of SiO gas to form Al-Si-O micron-size droplets, (**c-1**) VLS base growth of silica nanowire, (**d,a**) final silica nanowire; (**c-2**) VLS base growth of silica nanowire trunk, (**d-2**) formation of two Al-Si-O droplets on the top, (**e-2**) growth of branch-Y-shaped silica nanowire, (**f-2**) periodic stable growth of Y-shaped nanowire with direction changes, (**g-2**) growth of nanowire-ring structure, (**h-2**) final silica interconnected nanoring.

These results indicate that the growth process of the amorphous silica nanostructures in our study could be explained by vapor-liquid-solid (VLS) mechanism, which has been put forward by Wagner and Ellis^34^. Herein, Al(NO_3_)_3_ serves as the catalyst precursor and Al plays an important role in the growth and formation of the novel silica nanostructures, which was observed at the end of the nanowires by EDS spectra. A growth model for the silica nanostructures was proposed and shown in Fig. 5. The growth process could be presented in three steps. The first step is the formation of Al catalyst. Aluminum nitrate resolves at 135 °C, and converts into aluminium oxide. At higher temperature, the alumina might be decomposed into gaseous metallic Al and oxygen^35^. The Al atoms coalesce to nanoparticles, then deposit on the graphite paper and finally combine into micro-particles with high catalytic activity for the growth of silica nanostructures. The second step is the formation of gaseous silicon species. It is apparent that the silica is reduced to gas phase silicon monoxide and silica by carbon black, which also enables the formation of silica nanostructures by the VLS mechanism. The third step is the formation of silica nanostructures, including the nucleation step and growth step. Theoretically, the nucleation process could be further divided into monocentric and polycentric nucleation. When the gaseous silicon and silicon monoxide transport to the surface of the graphite paper and react with Al to form Al-Si-O droplets, the monocentric nucleation sites obtain firstly around the catalyst droplets. Simultaneously, both the silicon and silicon monoxide are oxidized to amorphous silica by the oxygen remained in the furnace or released from the refractory lining. As a result, the silica NWs begin to form, while the catalyst particles are lifted up from the graphite paper exactly as the normal VLS mechanism said. If the silica trunks periodically grow stably during the growth process, the silica nanowires would obtain on the graphite paper. But on the other hand, some Al-Si-O nanoparticles may also deposit on the top surface of the nanowire trunks (or one Al-Si-O droplet separates into two smaller Al-Si-O droplets) during the periodic stable growth, which would form two or more new growth centers (namely polycentric nucleation) and result in the branch-Y-shaped nanowires. The branch of Y-shaped nanowires can also grow comparable stably, but their growth directions may change and tend to from a ring. The change of growth direction may originate from the dislocation inside the nanostructures, but the growth mechanism of such a nanowire-ring formation process is still not clear. A possible explanation may be the effects of Van der Waals interactions or the surface energy minimization^23, 36, 37^. When the top of the branched nanowires meet together, the Al-Si-O catalytic droplets combine to become one new growth center. Then the next immediate procedure is another stable growth of silica nanowires. If the polycentric droplets were formed in the further reaction procedure, the nanowire-ring structure would be acquired again due to the polycentric nucleation and periodic stable growth with the gradual direction changes. Finally, the silica NWs and ICNRs products were obtained.

**Figure 6:**
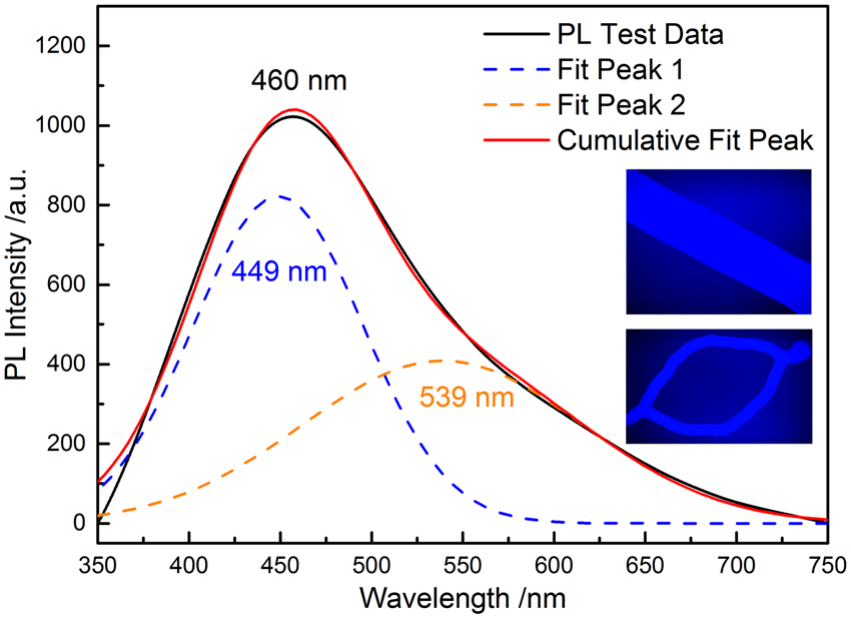
The PL emission spectrum and deconvolution fitted spectra of the as-obtained silica nanostructure products.

In order to investigate the optical properties of the novel silica nanostructures, the PL spectrum was recorded at room temperature using a Xe lamp as the excitation source. Figure 6 shows the PL emission spectrum of the as-prepared samples, excited at 254 nm (4.88 eV). The amorphous silica nanostructures exhibit a strong blue emission centered at 460 nm with a shoulder at long wavelength region. Therefore, we decompose the emisison spectrum into two well-fitted Gaussian peaks centered at 449 nm (2.76 eV) and 539 nm (2.30 eV). It has been reported that the 2.76 eV emission peak originates from the neutral oxygen vacancy (≡Si-Si≡), resulted from the reducing environment during the reaction procedure^38^. Thus the PL process could be discribed as follow: silica nanostructures firstly absorb the energy of 4.88 eV photons by ground-to-singlet transition or energy transfer from excited states (for example, the exictons at oxygen vacancy), then then the energy is tranferred from the excited singlet to the triplet states by intersystem crossing, and finally emit the 2.76 eV emission by forbidden transition from the triplet state to the singlet state^38, 39^. However, the emission at 2.30 eV observed in this study is scarely reported and the exact mechanism of the green emission for silica nanostructures also remains unclear^22^. The possible reason may be the rediative recombination of the selftrapped excitons, such as *e-h* pairs or excitons formed by the two-photon cross band-gap excitation^40^. Another possibility might be ascribed the surplus oxygen in local context of the amorphous silica products^38^. According to the recent literature, the PL properties of silica nanowires are different due to their various structures and stoichiometry^41^. The novel amorphous silica NWs and ICNRs here could be potential optical materials for visible light region.

## Discussion

Novel silica nanowire (NWs) and interconnected nanorings (ICNRs) have been successfully prepared via a typical thermal catalytic chemical vapor deposition (CVD) route using SiO_2_ powders and coke powders as raw materials. The as-prepared silica nanostructures mainly present as the morphologies of NWs and ICNRs, which are both about 200 nm in diameter. The structure investigations indicate that these nanostructures are amorphous. The growth of the silica nanowires are related to the normal vapor-liquid-solid (VLS) mechanism with monocentric nucleation and periodic stable growth process. While the growth process of silica interconnected nanorings are in accordance with the polycentric nucleation and periodic stable growth with the gradual direction changes. The PL measurement indicates that the strong blue emission spectrum for the as-synthesized silica nanostructures may be resulted from the combination of neutral oxygen vacancy (≡Si-Si≡) and selftrapped excitons. This study provides a simple method to gain new morphology for silica nanostructures, and the obtained novel silica NWs and ICNRs with outstanding optical properties could be considered as candidates for the application in optoelectronic nanodevices and light-emitting diode (LED) materials.

## Methods

**Figure 7:**
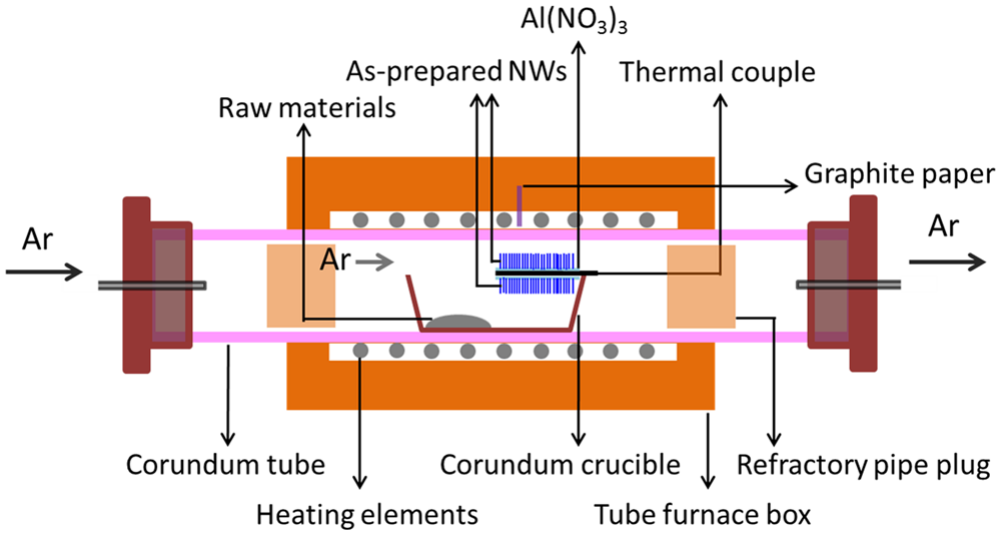
Schematic experimental setup for the synthesis of silica interconnected nanorings.

The typical synthesis reaction was conducted by a simple CVD procedure in a corundum tube furnace, using argon (purity 99.99%) as a carrier gas. The experimental setup is shown in Fig. 7. The starting materials, SiO_2_ powders and coke powders, were well-mixed in an agate mortar for 30 min. About 2 g of the milled mixtures was loaded in one side of a corundum crucible, and a graphite paper was placed on the opposite side dispersed with several drops of Al(NO_3_)_3_ aqueous solutions on both the top and bottom surfaces and dried in air. The aluminum crucible was put into the center of the long corundum tube in a tube furnace. The mixed powders were placed close to the argon-intake end. The experimental tube was vacuumized firstly by a rotary pump and then argon was introduced and kept flowing with a furnace pressure of 0.15 MPa during the whole heat treatment process. Meanwhile, the setup was heat from room temperature to 1350 °C at 3 °C/min, held for 3 h, and then cooled to room temperature naturally. Two white layers of products were finally obtained on both two sides of the graphite paper.

The as-prepared samples were characterized by field emission scanning electron microscopy (FESEM, Hitach S4800, Japan) and transmission electron microscopy (TEM/HRTEM, FEI-Tecnai-G^2^-F30, USA) equipped with energy dispersive X-ray spectroscopy (EDS). Fourier transform infrared spectroscopy (FT-IR) data were collected by a Nicolet IR100/200 spectrophotometer from 450 cm^−1^ to 1500 cm^−1^, using the standard KBr pellet technique. A PHI 5300 X-ray photoelectron spectroscopy system (XPS, Perkin Elmer, USA) was used to acquire the chemical states of Si 2p, and O 1s with a chamber base pressure of approximately 10^−7^ torr using Mg Kα X-rays at a take-off angle of 45°, and the total power was of 400 W. The PL spectra of as-prepared samples were recorded by fluorescence spectrophotometer (F-4600, Hitachi, Japan) using a 150 W Xe lamp as excitation source. All the measurements were determined at room temperature.
